# Exploring the Effectiveness of Combining Social Skills Training and Two Parent Programs in Improving the Social Competence of Children with Attention-Deficit/Hyperactivity Disorder

**DOI:** 10.3390/children12020132

**Published:** 2025-01-26

**Authors:** Rosa García-Castellar, Desirée Sánchez-Chiva, Belén Roselló-Miranda, Patricia Flor-Arasil

**Affiliations:** 1 Department of Developmental, Educational, Social and Methodological Psychology, University of Jaume I, 12006 Castellón de la Plana, Spain; 2 Faculty of Health Sciences, International University of Valencia, 46002 Valencia, Spain; desiree.sanchez@professor.universidadviu.com (D.S.-C.); patricia.flor@professor.universidadviu.com (P.F.-A.); 3 Department of Developmental and Educational Psychology, University of Valencia, 46010 Valencia, Spain; m.belen.rosello@uv.es

**Keywords:** ADHD, chat room task, SSRS-C, social skills training, parenting programs

## Abstract

**Background/Objectives**: The objective of the present study was to examine the extent of effectiveness of two parent programs to complement an intervention in social skills for children with attention-deficit/hyperactivity disorder (ADHD). **Methods**: Fifteen children with ADHD participated in a program to develop social skills, while their parents were randomly assigned to two parent training programs that had different formats: a Parenting School (N = 8) or a Parent Workshop (N = 7). There were no significant differences between parents of both groups in knowledge about ADHD, obedience to commands, or strategies for addressing desirable and undesirable behaviors. The pre- and post-intervention social cognition and social interaction of ADHD children were assessed using a SSRS-C questionnaire (Social Skills Rating System—Child Form) and a controlled chat room, which allowed for observing how children interact in a virtual environment. **Results**: Children showed significant improvements in social competencies, such as identifying social cues, generating appropriate responses, and reducing hostile responses, with large effect sizes, following the social skills intervention. Comparing the two parent training programs, the Parent Workshop program demonstrated significant improvements in social cue detection and conversation memory, with more children achieving reliable changes in these variables than in the Parenting School program. **Conclusions**: Finally, the reliable change indices for children showed that the Parent Workshop demonstrated improvements in more than half of the subjects across all analyzed variables. Study limitations, implications for research and practice are discussed.

## 1. Introduction

Approximately 7.7% of children aged 12 and under have attention-deficit/hyperactivity disorder (ADHD) [1]. This disorder is characterized by significant difficulties in maintaining attention and controlling hyperactivity and impulsivity [1]. In addition, problems in social functioning are generally associated with the disorder [2]. Children with ADHD show disruptive and inappropriate behaviors [3], and their social interaction is not characterized by reciprocity or empathic behaviors, such as supporting, consoling, sharing, seeing things from the other person’s point of view, or negotiating [4]. Furthermore, they are often unaware of their lack of social skills and, although they initiate interactions with peers, their efforts are often viewed as immature and intrusive [5]. Unfortunately, social problems in ADHD lead to adverse outcomes throughout life, increasing the risk of cigarette smoking, depression, anxiety, and global maladjustment [6].

Social skills involve different aspects of cognitions, emotions, and behaviors. Generally, children with ADHD are usually instructed in basic social norms, which focus on how to learn to wait their turn, interact appropriately in a conversation (that is, identify the right time to change the subject), and recognize the emotional expressions of other people. A systematic review by Willis et al. [7] suggested that social skills training constitutes a promising approach to improve the social functioning of children and adolescents with ADHD. Another more recent review by Storebo et al. [8], which included 45 trials, found that participants, their parents, and teachers in general expressed positive satisfaction with the intervention. In contrast, the perceived benefits of social skills training were limited and questionable due to the low certainty of the evidence, as half of the trials included in the review were at high risk of bias.

From an evolutionary perspective, in addressing the treatment of social deficits in childhood, the involvement of the family as a primary agent of development is necessary. Parents of children with ADHD make use of less-effective parenting styles that have been related to the intensity of ADHD symptomatology, as well as social difficulties associated with the disorder [9,10,11]. Therefore, the inclusion of parent training, incorporating positive parenting and behavioral management strategies, can be essential to facilitate the generalization to daily life of the social skills that children have learned. In fact, some relevant studies suggest the important role that parent training plays in interventions aimed at improving the social competence of children with ADHD. Jijina and Sinha [12] designed, to promote the generalization of learning, a program consisting of eight social skills training sessions with children with ADHD and the simultaneous participation of parents. The authors reported significant improvements in the scores of indicators of social functioning in the post-training assessment. Storebo et al. [13] also combined social skills training and work sessions with groups of parents in which the topics being worked on with the groups of children were discussed. Unlike Jijina and Sinha [12], the results indicated that the combination of social skills training plus a parental group was not more effective compared to the standard treatment. Therefore, an objective for future research would be to try to identify the most suitable structure and components of programs with parents to enhance the social competence of children with ADHD.

Work with families of children with ADHD has generally been developed from two different perspectives: Parent Workshops and Parenting Schools. Parent Workshops focus on specific interventions to improve parents’ coping skills and reduce symptoms in children. Their goal is to strengthen parents’ coping styles and promote their general well-being. This approach is also associated with significant improvements in both parents’ mental health and reductions in other frequent ADHD symptoms, such as ‘Social problems’, ‘Attention problems’, ‘Aggressive behavior’ or ‘Externalizing behavior’ [14]. Parenting Schools have a broader approach that includes educational components, often with an indirect impact on ADHD. They combine educational strategies, and their main purpose is to enhance global educational results. These interventions are generally not related to an improvement in the central symptoms of the disorder, but underlying results are given in the fact that parents allude to a possible effect on the symptoms of hyperactivity, and on the mental health problems that they themselves experience [15].

In summary, both types of programs (Parent Workshops and Parenting Schools) are designed to support families of children with ADHD, but each of them has some differential characteristics. In particular, Parent Workshops are more concrete and more structured than Parenting Schools, with sessions of a more practical nature. Therefore, analyzing both separately would identify best parent training practices to improve children’s social functioning across different domains. It would also be essential to consider the variability in individual children’s responses to the two programs for clinical or educational decision-making, as well as adjusting future intervention strategies.

The objective of the present study, which has an essentially exploratory character, was to examine the extent to which two parent programs—a Parenting School and a Parent Workshop—complement an intervention in social skills for children with ADHD. Furthermore, the most original contribution of this research consisted of trying to identify the relative therapeutic strength of each of the two programs developed with the parents. At least to our knowledge, up to now, no works have been published that raise this question. More specifically, this study aimed (1) to evaluate the changes in children’s social behavior after the intervention in social skills; (2) to compare the effects of both parent programs on children’s social behavior; and (3) to explore whether the Parenting School versus the Parent Workshop had a different impact on the Reliability Change Index in children’s social behavior [16].

## 2. Materials and Methods

### 2.1. Participants

The participants were fifteen Spanish children between 8 and 12 years of age (M = 9.60, SD = 1.121) and their families. All children had clinical diagnoses of ADHD by a healthcare professional, and they were receiving pharmacological treatment (generally psychostimulants). No children had psychosis, neurological damage, or sensory or motor deficits. Copies of the medical reports were provided by the parents to the study team.

To confirm the diagnosis of children with ADHD-C, parents were asked to complete the questionnaire based on the diagnostic criteria set out in the DSM-5 [2], based on the presence of at least six of nine inattention and six of nine hyperactive/impulsive symptoms. Children had a full-scale IQ score of 75 or more, as estimated by the Vocabulary and Block Design subtests on the Spanish version of the Wechsler Intelligence Scale for Children, Third Edition-Revised [17].

**Figure 1:**
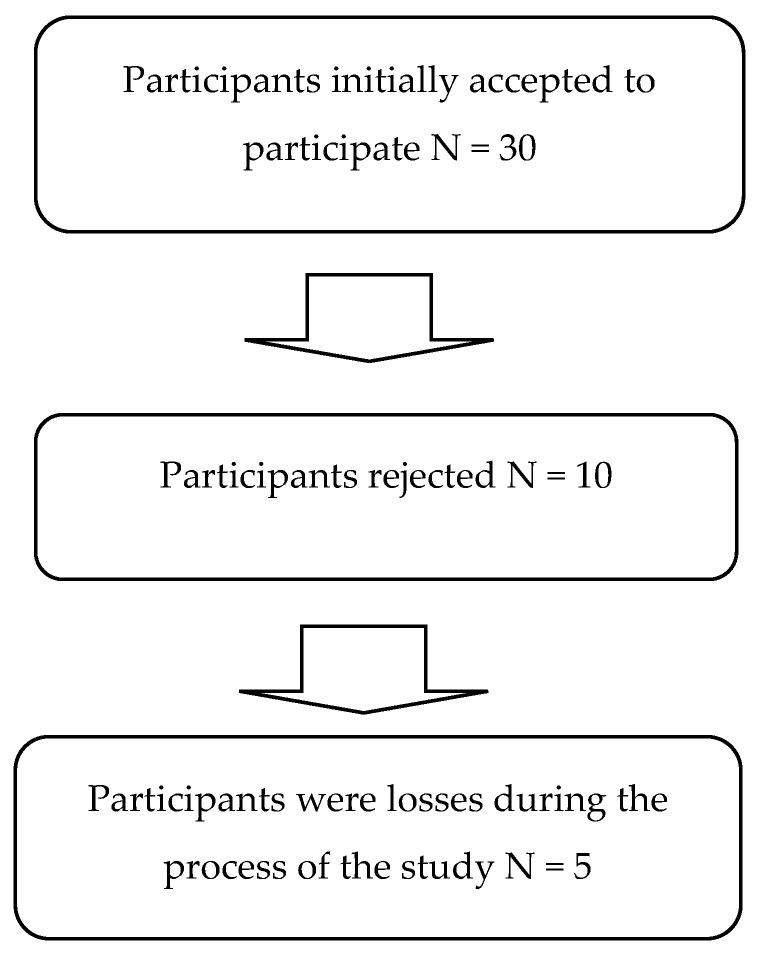
Recruitment of participants.

Originally, as shown in Figure 1, N = 30 participants committed to participate in the intervention program. However, 10 of them were excluded for not meeting any of the inclusion criteria specified above. Additionally, five families had to drop out of the study before completing the process.

The sample of 15 families that participated in the study were randomly assigned to one of two conditions: eight parents joined the Parenting School program group (SS/CH + PS), and seven parents participated in the Parent Workshop program group (SS/CH + PW). It was found that there were no differences between parents who were assigned to the Parenting Workshop or the School programs in the responses to three thematic blocks of the questionnaire on basic knowledge on ADHD: (1) Knowledge about ADHD *U* (15) = 23.00, *p* = 0.613; (2) Criteria for obeying orders *U* (15) = 24.50, *p* = 0.694; and (3) Actions against desirable and undesirable behaviors *U* (15) = 24.00, *p* = 0.694.

The mean age of eight children with ADHD in the social skills training plus Parenting School group (SS/CH + PS) was 9.13 ± 0.83 years, and 12.5% were girls. The mean age of seven children with ADHD in the social skills training plus Parent Workshop group (SS/CH + PW) was 10.14 ± 1.21 years, and 12.5% were girls. There were no differences between groups in terms of age (*t* (14) = 3.662, *p* = 0.078), full-scale IQ (*t* (14) = 0.367, *p* = 0.555), reading comprehension (*t* (14) = 1.203, *p* = 0.293) and typing skills total time in seconds (*U* (15) = 20.000, *p* = 0.355).

All the children with ADHD in this study were drawn from the sample used in [18]’s doctoral thesis, which examined various aspects of ADHD.

### 2.2. Description of the Intervention

Children Social Skills Program (SS/CH). The social skills program for children that was implemented was designed to address the primary cognitive and emotional deficits manifested by children with ADHD, combining different techniques, theories, models and activities: the Program for Teaching Social Interaction Skills (PEHIS) [19]; the Tough Kid Social Skills Program [20]; and the Social Skills in Childhood Program [21]. These programs are framed within a cognitive–behavioral approach and include multiple social skills. All of them contain different sessions in which examples are presented. In addition, practical exercises are carried out for teaching social skills to children with social interaction problems through instructional techniques such as modeling, role-playing and feedback.

The sessions for children were held one day a week for ninety minutes, over a period of 8 weeks. The following contents were worked on: (a) Basic social skills, (b) Emotions, (c) Conversational skills, (d) Self-control, (e) Assertiveness, and (f) Conflict resolution. Participants used role-plays, exercises, and games. These showed the required skills and were given positive or corrective feedback accordingly.

Parenting Intervention. The ‘Parent Workshop’ Intervention (PW) was developed in eight sessions with the following content: (a) Knowledge of ADHD (b) Social competencies (c) Self-esteem in parents, (d) Self-esteem in children (e) Positive communication, (f) Problem solving, (g) Increase positive behaviors, and (h) How to reduce inappropriate behaviors.

The ’Parenting School’ Intervention’ (PS) was also carried out over eight sessions, which included the following content: (a) Knowledge of ADHD, (b) The therapeutic approach to ADHD. False myths (c) Positive communication, (d) How to reduce inappropriate behaviors (e) Rules at home, (f) Parental self-control, (g) Parental responsibility and routines, and (h) Adolescence and adult life.

Both parenting programs included common content, namely awareness of ADHD, positive communication, and how to reduce inappropriate behaviors. However, in other sessions, the Parenting School focused on aspects related to norms and routines, while the specific sessions of the Workshop worked on more socio-affective content (social competencies, self-esteem of parents and children, increasing positive behaviors). Finally, the activities in the two parent programs were conducted individually, in pairs and in groups, to enrich the group with the advantages provided by different learning styles.

### 2.3. Measures

Reading Comprehension. In the present study, given that interaction in the chat required children to read text, it was essential to evaluate and control the participants’ reading ability. For this purpose, the text comprehension subtest of the Reading Processes Assessment Battery, Revised (PROLEC-R) [22], was administered. The text comprehension subtest of the PROLEC-R comprises four texts varying in length (90–130 words) and type (expository and narrative). A correct response was awarded with one point.

Typing Skills. Since the task in the chat required typing, it was critical to assess the speed and level of familiarity of the children with the keyboard. To measure typing speed, they were asked to transcribe three sentences dictated by the examiner. The time needed to complete the transcription of the three sentences was automatically recorded by the computer in milliseconds. The reliability calculated using Cronbach’s alpha was 0.72.

Social Skills. We employed the computerized chat room task used by Mikami et al. [23], adapted into Spanish [24]. This task was selected for its ability to provide a controlled, interactive environment that accurately reflects real-world social interactions and faithfully simulates the type of communication increasingly prevalent in contemporary society. Additionally, we used the Social Skill Rating System for Children self-questionnaire (SSRS-C) by Gresham and Elliot [25] adapted also into Spanish by the authors of the present paper.

The chat room task consists of simulating a virtual conversation between five friends in a chat to organize a birthday party. The child with ADHD must interact with the other four virtual children, whose dialogues are generated by the program. During the task, participating children have to read the conversation of the virtual friends, and the program is programmed to encourage the child to join the conversation and interact with them. The conversation in the chat room lasts about 10 min. At the end, the children are asked questions about the conversation they have had in the chat room. In addition, the application provides a transcript of the conversation, in which the answers of the participants can be seen, and the reaction times in which these responses are produced are collected. We highlight that the program replicates the same conversation between children for each one who participates; this provides a controlled stimulus to which participants can respond in unique ways.

Following Mikami et al. [23], the following task variables were collected: the detection of social cues, generation of relevant responses, elaboration of responses, prosocial responses, and hostile responses. First, in social cue detection, it is assessed whether the child detects key cues from peers to ask questions about certain topics. Second, in generating relevant responses, we assess whether the responses are adequately related to the topic of the conversation. Thirdly, in the preparation of responses, the degree of elaboration of each participant’s response is evaluated. Fourthly, the prosocial responses that the child provides during the conversation are collected. Fifth, hostile responses are counted for conflicting responses provided by participants during the conversation. And finally, conversational memory is measured. The test–retest reliability of the chat room task was Pearson’s r = 0.0532 [24].

The Social Skill Rating System for Children (SSRS-C) questionnaire by Gresham and Elliot [22], the present instrument, provides information on the frequency and importance of Cooperation, Assertiveness, Self-control and Empathy. This questionnaire is made up of 39 items, which are classified with a three-point Likert scale, ranging from a score of 0 (never) to 2 (frequently). Therefore, the maximum score that can be obtained is 78.

The SSRS-C was adapted from Sánchez [18] and used to assess the self-perception of social skills of children with and without ADHD. The internal consistency of the SSRS subscales ranged from 0.51 to 0.91, with an internal consistency of 0.75. In the version adapted to Spanish, the authors found high Cronbach’s alpha coefficients (0.89) for the Spanish adaptation.

Questionnaire for Parents on Basic Knowledge of ADHD. This is a brief questionnaire specifically designed by the authors for the research. It comprises three content sections on ADHD: (1) Knowledge about ADHD (twelve items) ‘I consider that the child with ADHD, in addition to medication, needs psychological support’; (2) Criteria for following instructions (six items) ‘I usually give several orders at the same time’; and (3) Actions addressing desirable and undesirable behaviors (seven items) ‘I think the hyperactive child needs more praise and positive reinforcement than may other children’. The response options are ‘yes’ or ‘no.’ Higher scores indicate greater knowledge about ADHD.

### 2.4. Procedure

The present study was conducted in the province of Castellón (Spain). The children with ADHD and their families were contacted through the Association of Parents affected by Attention Deficit Disorder of Castellón ‘APADAHCAS’. All families provided written consent to study procedures, which were approved by the Ethical Committee of the University, reflecting the ethical principles for research with human beings. Parents gave consent at the same time for their children to participate.

The children’s assessment process took place at school, where they supplemented the study measures in individual soundproof test rooms under the supervision of an examiner. For children with ADHD, two assessment sessions were usually scheduled, each lasting about 40 to 60 min, because of their difficulties with attention and behavior.

The social skills intervention sessions for children took place in a research laboratory at the Jaume I University of Castellón, performed by a psychologist from our research team. Each session lasted 90 minutes and was carried out once a week. The Parent Workshop and Parenting School intervention sessions also took place at Jaume I University over 8 weeks. On the other hand, the Parenting School intervention sessions were held on Saturday mornings and Parent Workshop intervention sessions were held on Friday afternoons. The same psychologist responsible for the intervention with children developed the sessions with parents, recording whether the proposed activities were being implemented at home.

### 2.5. Statistical Analysis

The data were analyzed using IBM SPSS Statistics, version 29.0, with a significance level set at *p* < 0.05 for all tests. The Shapiro–Wilk test was applied to all the study variables to verify that the data distribution met the statistical normality criterion (Shapiro–Wilk *p*-values > 0.05). The continuous variables were summarized as medians and interquartile ranges. The Student’s *t*-test, Wilcoxon Signed-Rank test and U Mann–Whitney test were used, respectively. The effect size for comparation groups was computed using Cohen’s *d* or Rosenthal’s *r* and interpreted—based on the following metrics: small = 0.2; medium = 0.5; large = 0.8—to evaluate the magnitude of the observed effects.

## 3. Results

### 3.1. Effects on the Social Competencies of All Children After the Intervention in Social Skills

The intragroup comparisons of children with ADHD between pre-test and post-test, considering the participation of parents in the intervention programs, showed statistically significant differences in all the chat room variables (Table 1).

However, as shown in Table 2, in the analysis of SRSS-C self-report, no significant differences were observed between pre-treatment and after the intervention in social competences, with the exception of the assertiveness variable.

### 3.2. Effects Between Groups of the Complementation of the Parenting School Versus Parent Workshop Programs on Children’s Social Competencies After the Intervention in Social Skills

The comparison in the chat room task in the pre-test between the group of children with ADHD whose parents had attended the Parenting School program and the Parent Workshop program group did not show significant differences in any of the analyzed variables: Detection of social cues (t = 1.147; *p* = 0.272), Generating on-topic responses (t = 2.120; *p* = 0.054); Elaboration of response (t = 1.374; *p* = 0.193); Prosocial responses (z = −0.634, *p* = 0.526); Hostile responses (z = −0.069, *p* = 0.945) and Conversational memory (t = −0.025; *p* = 0.490).

As shown in Table 3, the comparative analysis between the two programs demonstrated that the Parent Workshop in the post-intervention achieved significant improvements in most of the variables, reaching high effect sizes: Detection of social cues (Cohen’s *d* = 0.83), Generating on-topic responses (Cohen’s *d* = 0.84), Prosocial responses (Rosenthal’s *r* = −0.58) and Conversational memory (Cohen’s *d* = 0.9).

### 3.3. Analysis of the Reliability Change Index in Children’s Social Competencies According to Parental Intervention (Parenting School Versus Parent Workshop)

The effectiveness of treatment in social skills was analyzed individually comparing the results of the two programs for parents using the chat room task. The analysis focused on those variables that showed statistical significance between the pre-test and post-test measurements, and that also met the requirement of a normal distribution. The Reliability Change Index (RCI) was used [16].

**Figure 2:**
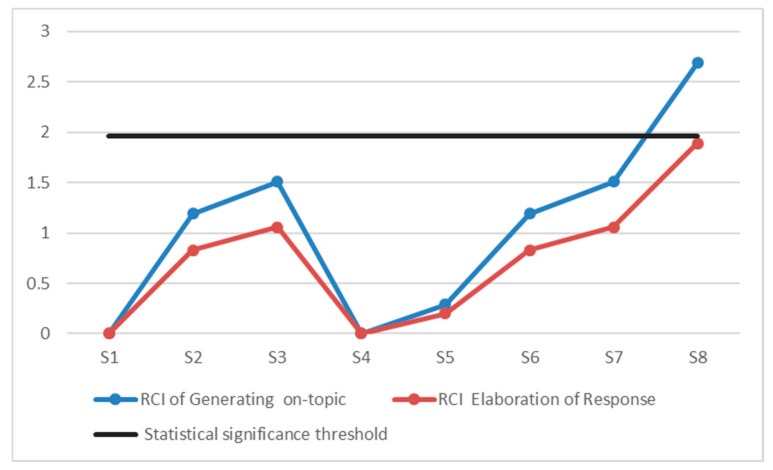
Representation of the Reliable Change Indices (RCI) of the eight children whose parents attended the ’Parenting School’ Intervention.

In the Parenting School intervention, significant differences were found between the pre-test and post-test measurements in the following variables: Generating on-topic responses (t (7) = −2.662, *p* = 0.016); Cohen’s *d* = 0.29 and Elaboration of response (t (7) = −3.157, *p* = 0.008); Cohen’s *d* = 0.41. As shown in Figure 2, RCI analysis for Generating on-topic responses showed that, of the eight children, only one child (RCI ≥ 1.96) presented a significant reliable change. On the contrary, in the Elaboration of response variable, no child achieved a significant reliable change.

In the Parent Workshop intervention, significant differences were found between the pre-test and post-test measurements in the following variables: Detection of social cues (t (6) = −2.763, *p* = 0.016); Cohen’s *d* = 0.16, Generating on-topic responses (t (6) = −4.054, *p* = 0.003); Cohen’s *d* = 0.15, Elaboration of response (t (6) = −4.176, *p* = 0.003); Cohen’s *d* = 0.37 and Conversational memory (t (6) = −5.284, *p* < 0.001); Cohen’s *d* = 3.14.

**Figure 3:**
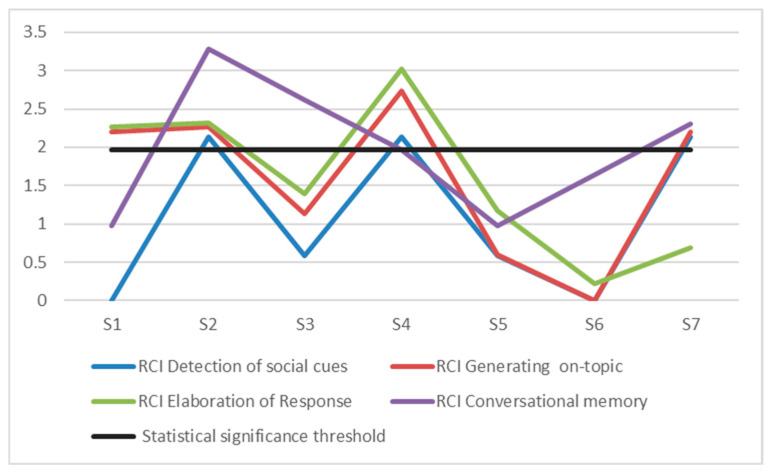
Representation of the Reliable Change Indices (RCI) of the seven children whose parents attended the ’Parenting Workshop’ Intervention.

As shown in Figure 3, the analysis of the Reliable Change Index (RCI) for the variable Detection of social cues revealed that, out of the seven children, three demonstrated a significant reliable change (RCI ≥ 1.96). For the variable Generating on-topic responses, four children showed a reliable change. In the Elaboration of response variable, three children exhibited a significant reliable change, and for the Conversational memory variable, four children showed a significant reliable change. Overall, the intervention in the Parent Workshop, combined with the social skills intervention for the children, resulted in reliable improvements, with 50% or more of the children demonstrating significant changes.

## 4. Discussion and Conclusions

The aim of this study was to evaluate changes in children’s social behavior following a social skills intervention, considering the simultaneous participation of parents in related programs. The results provide a nuanced understanding of the effects of such interventions.

The first objective of this study was to evaluate the changes in children’s social behavior after the intervention in social skills. The analysis of pre- and post-intervention measurements in children with ADHD demonstrated significant improvements across all variables evaluated in the chat room context, which involved engaging in conversations with avatars. These variables included key social interaction skills, such as detecting social cues, generating topic-related responses, elaborating complex responses, and producing prosocial responses, alongside a reduction in hostile responses. These findings indicate that children with ADHD show improvement after the intervention, even in later stages of social cue detection and encoding, which are known deficits in these children, as highlighted by previous research [23,24] using similar evaluation methodologies.

Although there is no consensus on a social cognitive profile or which skills predict behavior in children with ADHD, some studies suggest that working memory deficits may underline difficulties in social perception and affect their ability to interact and maintain social relationships [26]. Our results showed a significant improvement in conversational memory, suggesting that the intervention not only promoted direct social skills but also enhanced associated cognitive abilities, especially when realistic simulations of group conversations related to real-life situations were incorporated.

This improvement in conversational memory has practical implications, as it may help children with ADHD recall details of past interactions, follow ongoing discussions, and respond appropriately to social cues—key skills for forming and maintaining relationships. In school, these enhancements could enable more meaningful participation in group projects and decision-making, supporting both academic performance and social integration.

The results indicate no significant improvements in self-perceived frequency of use or importance of social skills such as empathy, cooperation, and self-control among children with ADHD following the intervention, except for a noted increase in the perceived importance of assertiveness. This finding highlights the importance of considering participants’ perspectives to assess the real impact of interventions—an aspect often overlooked in prior studies that have focused on adult perceptions [27]. Furthermore, research suggests that children with ADHD may exhibit positive illusory bias, leading to an overestimation of their social skills [28], or in some cases, an underestimation, particularly in contexts involving potential friendships [29]. Such variability in social self-perceptions presents significant challenges for intervention design, as both the overestimation and underestimation of social competencies can hinder meaningful change.

The lack of improvement might also relate to difficulties distinguishing between fluency deficits caused by self-control issues versus those stemming from low motivation or interest in assessment tasks, as suggested by earlier research [30]. Additionally, self-report methods may be limited by participants’ introspective abilities and self-awareness, especially in populations with ADHD [31]. It is plausible that perceived social skills require a longer intervention period or a broader contextual scope to manifest more tangibly in children’s self-perceptions, which may explain the observed results.

Regarding the second objective, comparative analyses between the groups of children whose parents participated in the Parent Workshop (PW) program and those who attended the Parenting School (PS) program yielded interesting results. In the pre-intervention assessment, no differences were found between the two groups, indicating that both parental programs started from a homogeneous baseline in terms of their initial impact on children’s social competencies.

However, post-intervention analyses revealed a significant improvement in the social competencies of children whose parents participated in the Parent Workshop program. These improvements were observed in key aspects such as Detection of social cues, Generating on-topic responses, Prosocial responses, and Conversational memory. The large effect sizes for these variables highlight that the intervention was not only effective but also had a substantial impact on the social development of children with ADHD. These large effect sizes not only show the effectiveness of the program but also provide information on how it can benefit children clinically and educationally. Since at the clinical level these improvements contribute to the development of social skills, in turn, they can reduce the difficulties associated with ADHD, facilitating better integration into social contexts. Regarding the educational field, children will improve these social skills and favor them in their relationship with classmates and teachers, having a more active and positive participation in their school environment. Nonetheless, no significant differences were identified between the two parental programs in terms of the children’s ability to elaborate responses (Elaboration of responses) or in the reduction of hostile responses. This may be explained by the attention-deficit characteristics of children with ADHD, which affect the more complex stages of information processing, such as the formulation of appropriate responses.

The combination of a social skills program targeted at children with the Parent Workshop (SS-CH + PW), which addresses specific aspects of social competencies more closely related to social performance rather than social knowledge—such as improving self-esteem in both parents and children and solving social problems—appears to generate greater benefits compared to a more general program like the Parenting School (SS-CH + PS). These findings align with previous research [12], which highlights that active parental involvement in specific interventions facilitates the generalization of skills learned by children to broader contexts, such as family and school environments.

The third objective of this study was to explore whether Parenting School versus Parent Workshop interventions had a different impact on the Reliable Change Index (RCI) in children’s social behavior. This approach provides a more precise evaluation of the differential impact of the interventions, enabling the identification of clinically significant changes rather than merely statistical differences. In the group of children whose parents participated in the Parenting School program, significant differences were observed between pre-test and post-test measurements in the variables Generating on-topic responses and Elaboration of responses, with moderate effect sizes. However, the RCI analysis revealed that for the variable Generating on-topic responses, only one child achieved a significant reliable change, and for Elaboration of responses, no child met this criterion. This suggests that, although the program was effective at the group level, its individual impact was limited. Such a result could be explained by factors such as the heterogeneity of the Parenting School program’s content or individual differences in children’s capacity to benefit from the more general approach applied in this parental program.

On the other hand, the group of children in the Parent Workshop program showed significant improvements across a greater number of chat room variables: Detection of social cues, Generating on-topic responses, Elaboration of responses, and Conversational memory. Effect sizes varied, but a particularly large effect was observed for Conversational memory. These results suggest that this program not only promoted improvements across a broader range of social skills but also had a notably strong impact on Conversational memory. This outcome could be attributed to the workshop’s more practical and intensive approach, which prioritized specific aspects such as self-esteem and social competencies, thereby facilitating the greater consolidation and transfer of social skills in children. The RCI analysis reinforces these findings, as more than 50% of the children whose parents attended the Parent Workshop demonstrated individual reliable improvements.

Our findings represent significant advancements in the field of social competencies in children with ADHD. Unlike previous studies that rely on standardized measures, we employed functional measures, such as the detection of social cues and response elaboration, which assess the impact of interventions in real-world contexts, providing a more interactive and contextual perspective. The findings of this study also highlight that specific parent training programs focusing on social skills yield more significant functional improvements than general parent training programs, suggesting that combined interventions for children and parents maximize therapeutic impact. Furthermore, the use of reliable change indices confirmed substantial improvements in more than half of the participants, advancing beyond the study by Wilkes-Gillan et al. [32], which did not evaluate effects using this methodology or compare different parent training programs. These findings underscore the importance of integrating functional measures, targeted parent training, and rigorous analyses to optimize interventions for children with ADHD.

In conclusion, this study highlights the importance of designing comprehensive interventions targeting both children and their caregivers. While both parental programs contributed to the development of social skills, the Parent Workshop demonstrated superior efficacy, particularly in consolidating key social competencies in children with ADHD. Additionally, it is especially noteworthy that the variable Conversational memory exhibited a particularly high effect size, suggesting that this type of intervention not only fostered specific social skills but also enhanced retention and cognitive processing abilities in social interaction contexts. Future research should explore how different formats of parental programs can influence the long-term sustainability of these outcomes and to what extent these findings can be generalized to other contexts or clinical populations.

Despite the promising findings, this study is not without limitations that must be considered. First, the small sample size limits the ability to generalize the findings to broader populations. Previous studies have noted that small sample sizes can influence effect sizes and reduce the reliability and robustness of statistical analyses [33]. Future studies with larger sample sizes are needed to validate and expand these findings. Second, the study evaluated changes only in the short term, without assessing whether improvements in social competencies were maintained over time. The literature on ADHD interventions indicates that positive effects may diminish without adequate follow-up strategies [34]. Incorporating longitudinal evaluations would help determine the sustainability of the observed improvements and provide insight into the long-term impact of intervention, guiding future program development. Third, although the study design included two parental intervention conditions, the absence of a no-intervention control group limits the ability to attribute the improvements exclusively to the implemented programs. Furthermore, statistically significant changes do not always translate into clinically meaningful improvements [16]. Finally, while objective measures and self-reports were used to assess social skills, the perspectives of parents, teachers, or peers were not included. Studies [35] have demonstrated that third-party evaluations are crucial for obtaining a more comprehensive understanding of the impact of interventions on the social behavior of children with ADHD.

## Figures and Tables

**Table 1:** Effect of the chat room task pre-/post-intervention.

Dimensions	Pre-Intervention				Post-Intervention				Effect Size (d)	*p*-Value
	n ADHD = 15				n ADHD = 15					
	Mean	SD	Mdn	IQR	Mean	SD	Mdn	IQR		
Detection of social cues	0.594	0.306	-	-	0.744	0.187	-	-	*d* = 0.246	t = −2.358, *p* = 0.017 *
Generating on-topic responses	0.427	0.251	-	-	0.683	0.186	-	-	*d* = 0.228	t = −4.338, *p* = < 0.001 **
Elaboration of response	0.805	0.528	-	-	1.322	0.617	-	-	*d* = 0.383	t = −5.219, *p* = < 0.001 **
Prosocial responses	0.038	0.045	0.000	0.08	0.236	0.163	0.166	0.250	*r* = 0.882	W = 120.000, *p* < 0.001 **
Hostile responses	0.044	0.070	0.000	0.80	0.000	0.000	0.000	0.000	*r* = −0.527	W = 0.000, *p* = 0.041 *
Conversational memory	9.5	5.289	-	-	13.2	5.417	-	-	*d* = −0.609	t = −2.358 *p* = 0.017 *

Abbreviations: (ADHD), Attention-Deficit/Hyperactivity Disorder; (t), Student’s t; (W), Wilcoxon Signed-Rank Test Probability; n, sample size; (Mdn), Median; (IQR), interquartile range, or Mean (SD), standard deviation; Effect sizes: Cohen’s d for continuous variables, odds ratios for categorical variables: Rosenthal, r; * *p* < 0.05; ** *p*< 0.01.

**Table 2:** Effect of the Social Skill Ranking System for Children questionnaire pre-/post-intervention.

Dimensions	Pre-Intervention		Post-Intervention			
	*n* ADHD = 15		*n* ADHD = 15			
	Mean	SD	Mean	SD	Cohen’s *d*	*p* Value
(F) Cooperation	13.4	4.611	13.13	2.669	3.91	0.796
(F) Assertiveness	14	3.162	13.93	3.535	3.63	0.944
(F) Empathy	14.47	3.889	14.27	3.127	3.50	0.828
(F) Self-Control	12.67	4.203	10.67	3.352	3.72	0.056
(I) Cooperation	13.27	13.27	13.27	4.828	2.84	0.929
(I) Assertiveness	11.20	4.617	12.80	3.932	2.29	0.017 *
(I) Emphaty	13.07	5.077	5.077	4.395	2.23	0.820
(I) Self-control.	13.53	5.768	12.8	5.321	2.71	0.313

(I), importance; (F), frequency; n, sample size; SD, standard deviation; Cohen’s d, effect size; Student’s *t*-test, * variable with significant effect (*p* < 0.05).

**Table 3:** Comparative post-intervention effect on both parents’ groups.

Dimensions	Post-Intervention					
	Parenting School Program (n ADHD = 8)		Parent Workshop Program (n ADHD = 7)		Effect Size *(d)/(r)*	*p* Value
	Mean (SD)	Mdn (IQR)	Mean (SD)	Mdn (IQR)		
Detection of social cues	0.645 (0.192)	-	0.857 (0.104)	-	*d* = 0.83	*p* = 0.023 * ^t^ (SS-CH + PS) < (SS-CH + PW)
Generating on-topic responses	0.583 (0.172)	-	0.797 (0.134)	-	*d* = 0.84	*p* = 0.020 * ^t^ (SS-CH + PS) < (SS-CH + PW)
Elaboration of response	1.090 (0.529)	-	1.580 (0.643)	-	*d* = 0.66	*p* = 0.130 ^ns t^
Prosocial responses	0.161 (0.140)	0.125 (0.08)	0.321 (0.147)	0.333 (0.25)	*r* = −0.58	*p* = 0.024 * ^Z^ (SS-CH + PS) < (SS-CH + PW)
Hostile responses	-	0.000 (0.00)	-	0.000 (0.00)	*r* = 0.01	*p* = 1.000 ^ns Z^
Conversational memory	11 (5.63)	-	15.85 (4.09)	-	*d* = −0.9	*p* = 0.041 * ^t^ (SS-CH + PS) < (SS-CH + PT)

Abbreviations: (SS-CH + PS), Program children plus ‘Parenting School’ Intervention; (SS-CH + PW), Program children plus ‘Parenting Workshop’ Intervention; (Mdn), Median; (IQR), interquartile range, or Mean (SD), standard deviation; ^t^: Student’s test; ^Z^: Z value in the Wilcoxon Signed-Rank test; Effect sizes: Cohen’s *d* or Rosenthal, *r*; * *p* < 0.05, ^ns^: not significant

## Data Availability

The raw data supporting the conclusions of this article are available at [http://hdl.handle.net/10234/704555], accessed on 11 December 2024.
